# Association of Residence in High–Police Contact Neighborhoods With Preterm Birth Among Black and White Individuals in Minneapolis

**DOI:** 10.1001/jamanetworkopen.2021.30290

**Published:** 2021-12-08

**Authors:** Rachel R. Hardeman, Tongtan Chantarat, Morrison Luke Smith, J’Mag Karbeah, David C. Van Riper, Dara D. Mendez

**Affiliations:** 1 Division of Health Policy and Management, University of Minnesota School of Public Health, Minneapolis; 2 Minnesota Population Center, Institute for Social Research & Data Innovation, Minneapolis; 3 Center for Antiracism Research for Health Equity, University of Minnesota School of Public Health, Minneapolis; 4 Division of Epidemiology and Community Health, University of Minnesota School of Public Health, Minneapolis; 5 Department of Epidemiology, University of Pittsburgh Graduate School of Public Health, Pittsburgh, Pennsylvania

## Abstract

**Importance:**

Police contact may have negative psychological effects on pregnant people, and psychological stress has been linked to preterm birth (ie, birth at <37 weeks’ gestation). Existing knowledge of racial disparities in policing patterns and their associations with health suggest redesigning public safety policies could contribute to racial health equity.

**Objective:**

To examine the association between community-level police contact and the risk of preterm birth among White pregnant people, US-born Black pregnant people, and Black pregnant people who were born outside the US.

**Design, Setting, and Participants:**

This cross-sectional study used medical record data of 745 White individuals, 121 US-born Black individuals, and 193 Black individuals born outside the US who were Minneapolis residents and gave birth to a live singleton at a large health system between January 1 and December 31, 2016. Data were analyzed from March 2019 to October 2020.

**Exposures:**

Police contact was measured at the level of the census tract where the pregnant people lived. Police incidents per capita (ie, the number of police incidents divided by the census tract population estimate) were dichotomized into high if the value was in the fourth quartile and low for the remaining three quartiles.

**Main Outcomes and Measures:**

Preterm birth status was based on the *International Statistical Classification of Diseases and Related Health Problems, 10th revision, Clinical Modification *(*ICD-10-CM*) code. Preterm infants were those with *ICD-10-CM* codes P07.2 and P07.3 documented in their charts.

**Results:**

Of 1059 pregnant people (745 [70.3%] White, 121 [11.4%] US-born Black, 193 [18.2%] Black born outside the US) in the sample, 336 White individuals (45.1%) and 62 Black individuals who were born outside the US (32.1%) gave birth between the ages of 30 and 34 years, while US-born Black individuals gave birth at younger ages, with 49 (40.5%) aged 25 years or younger. The incidence of preterm birth was 6.7% for White individuals (50 pregnant people), 14.0% for US-born Black individuals (17 pregnant people), and 5.7% for Black individuals born outside the US (11 pregnant people). In areas with high police contact vs low police contact, the odds of preterm birth were 90% higher for White individuals (odds ratio [OR], 1.9; 95% CI, 1.9-2.0), 100% higher for US-born Black individuals (OR, 2.0; 95% CI, 1.8-2.2), and 10% higher for Black individuals born outside the US (OR, 1.1; 95% CI, 1.0-1.2). Secondary geospatial analysis further revealed that the proportion of Black residents in Minneapolis census tracts was correlated with the number of police incidents reported between 2012 and 2016 (*P* = .001).

**Conclusions and Relevance:**

In this study, police contact was associated with preterm birth for both Black and White pregnant people. Predominantly Black neighborhoods had greater police contact than predominantly White neighborhoods, indicating that Black pregnant people were more likely to be exposed to police than White pregnant people. These findings suggest that racialized police patterns borne from a history of racism in the United States may contribute to racial disparity in preterm birth.

## Key Points

**Question:**

Is living in a neighborhood with high police presence associated with increased risk of preterm birth?

**Findings:**

In this cross-sectional study of 1059 Minneapolis residents who gave birth to a live singleton in 2016, the odds of preterm birth for pregnant people living in a neighborhood with high police presence was significantly higher compared with the odds of their racial counterparts in a low-presence neighborhood (90% increase for White individuals, 100% increase for US-born Black individuals, and 10% for Black individuals born outside of the US). The higher the proportion of Black residents in the neighborhood, the greater the number of police incident reports.

**Meaning:**

These findings suggest that greater police presence in Black vs White neighborhoods may contribute to the persistent Black-White preterm birth disparity in Minneapolis.

## Teaser

This cross-sectional study examines the association between community-level police contact and the risk of preterm birth among White pregnant people, US-born Black pregnant people, and Black pregnant people who were born outside the US.

## Introduction

Black pregnant people in the United States experience preterm birth (PTB; birth at <37 weeks’ gestation) at rates approximately 2 times that of White pregnant people.^1^ Black pregnant people are also twice as likely to experience the death of an infant younger than 1 year, a disparity primarily associated with preterm-related causes of death.^2^ These entrenched disparities remain unmoved by decades of public health research and persist despite increased access to prenatal care. They are not explained by differences in health behaviors, and they affect Black individuals of all socioeconomic statuses (SESs).^3^ The persistent and racialized nature of these inequities points to racism as a root cause.

Racism shows up in many ways in our society. One manifestation is residential racial segregation (ie, geographic separation by race). Black pregnant people who live in areas with high levels of racial segregation are more likely to give birth prematurely.^4^ Residential segregation relegates Black people to neighborhoods disproportionately affected by poverty, violence, and crime.^5,6^ In lieu of policy solutions to address these issues, greater police presence has been the answer in many communities—a practice known as proactive policing.^7^ Proactive policing has led to a policing system that attempts to aggressively maintain order by preventing crime before it occurs through what is commonly referred to as broken windows policing.^8^ It also encourages more frequent involuntary contact between law enforcement and the public. Due to socially and often racially biased notions of criminality and deviance, these policies have resulted in the disproportionate police presence in low-income and majority Black neighborhoods.^7^ In this study, we define overpolicing as the disproportionate targeting of Black communities by law enforcement that does not correlate to the incidence of crime within neighborhoods but rather reflects racist beliefs about Black deviance.^9,10^ Research has shown that men report increased psychological distress in neighborhood-level increases in aggressive policing (ie, frisking and use of force by police).^11^ However, to our knowledge, there is no literature that seeks to understand how aggressive policing and increased police surveillance affect maternal and infant health, specifically PTB. Recent evidence suggests that states with more killings of unarmed Black people by police have larger Black-White PTB disparities.^12^ Given that PTB is believed to be influenced by a wide range of environmental and psychosocial factors and their interactions,^13,14,15,16^ it is important to understand whether living in a community with a disproportionate amount of police presence affects the risk of PTB.

This study examined the association between police contact and PTB in US-born Black, Black born outside the US, and White pregnant people in Minneapolis, Minnesota. Minneapolis is a well-positioned geographic location for our study for several reasons. First, despite its reputation as a politically progressive city with forward-thinking urban planning, Minneapolis has some of the worst scores for residential racial segregation measures in the nation.^6^ Second, Minneapolis is home to an ethnically diverse Black population—notably one of the largest Somali communities outside of Somalia^17^—providing a unique opportunity to assess the intersectionality between race and nativity and its joint association with police contact patterns and racial inequity in PTB. Third, the stark disparity in PTB rates for Black and White pregnant people and infant mortality in Minneapolis has been well documented.^18^ As the city continues to reimagine public safety, particularly in response to the killing of George Floyd, Jr, results from this study will add to the body of evidence that will inform the redesign of public policies in Minneapolis and elsewhere in the United States.^19^

Leveraging patient medical records from a large health care system in the Minneapolis–St Paul area, detailed spatial data, and city-level policing data, we aimed to answer 2 questions: (1) is excessive police contact associated with increased risk of PTB for White pregnant people, US-born Black pregnant people, and Black pregnant people born outside the US in Minneapolis, and (2) is police contact racialized (ie, more police contact in Black than White neighborhoods) in a way that could suggest increased exposure and subsequent risk of PTB?

## Methods

### Study Cohort

Our study cohort included pregnant people who gave birth to live singletons from January 1 to December 31, 2016, at Fairview Health System facilities (8545 individuals). We further restricted our analytical sample to Black and White individuals (based on their self-reported race) whose residential address on the medical record fell within one of the 116 census tracts that make up the city of Minneapolis plus 15 bordering census tracts (1059 individuals). We included pregnant people from bordering census tracts because many police incidents reported in the Minneapolis Police database occurred in these areas based on the result of our geocoding, as discussed in the Police Contact section. We divided our cohort into 3 racial groups: White (745 pregnant people [70.3%]), US-born Black (121 pregnant people [11.4%]), and Black born outside the US (193 pregnant people [18.2%]) (eFigure in the Supplement).

This study was designated as non–human subject research by the institutional review board of the University of Minnesota, and therefore, informed consent was waived. This cross-sectional study adhered to the Strengthening the Reporting of Observational Studies in Epidemiology (STROBE) reporting guideline.^20^

### Outcome

PTB was defined using the *International Classification of Diseases, 10th revision, Clinical Modification *(*ICD-10-CM*) codes. The diagnosis code P07.2 (extreme immaturity of a newborn) and P07.3 (preterm [premature] newborn [other]) documented in the infant medical records indicated that the infant was born before 37 weeks of gestation.

### Police Contact

**Figure 1.**
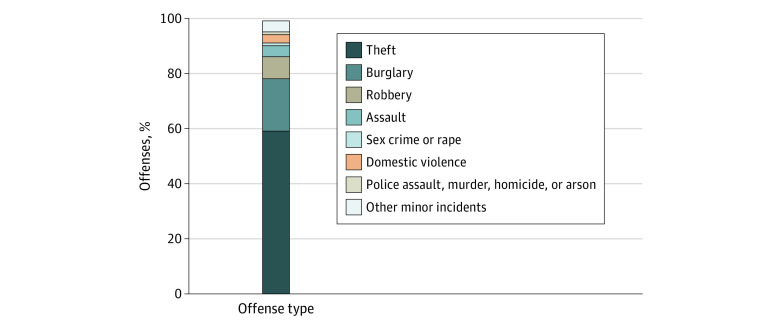
Types of Offenses Reported in the City of Minneapolis Police Incident Report, 2012 to 2016

We operationalized and measured community-level police contact at the census tract level, using the police incident data from the City of Minneapolis Police Incident Report.^21^ This database contained information on the date and time the incidents were reported by police officers, the type of offense (Figure 1), and the geographic coordinates of the incidents. These incidents do not represent all contacts between police and members of the public, only those for which police officers filed official reports and to which uniform crime reporting coding was assigned. To ensure that we captured the extent of police contact from all Minneapolis census tracts, we pooled police incident reports from 2012 to 2016 and assigned a census tract identifier to each incident. The police incidents reported in this database were not precisely located on a street boundary. When we intersected the small buffers with the census tracts, buffers that appeared to be on the border of Minneapolis were assigned census tract identification for 15 tracts located outside Minneapolis.

The 5-year cumulative count of police incidents of all offense types was calculated for each census tract. For this period, the value ranged from 10 to more than 9000 incidents. To account for increased incidents due to a larger census tract population size, we calculated per-capita police contact by dividing the cumulative count of police incidents in each census tract by the census tract population estimate from the 2012-2016 American Community Survey (ACS) 5-year estimates.^22^ Based on the per-capita police contact from 116 Minneapolis and 15 bordering census tracts, we dichotomized census tracts into those with high (fourth quartile) vs low (first to third quartile) police contact. For sensitivity purposes, we also assigned census tracts’ police contact status based on tertiles (ie, third tertile vs first and second tertiles). This tertile-based measure put individuals who lived in a census tract with moderately high police contact (ie, from the 67th to 74th percentile) in the high exposure group. Hence, census tracts with police contact per capita in the third tertile and those in the fourth quartile may be treated as high and very high exposure neighborhoods, respectively.

### Neighborhood Racial Composition

We extracted the number of residents identified as Black or African American to derive the proportion of Black residents in all Minneapolis and neighboring census tracts. The number of residents identified as Black or African American was based on the 2012-2016 ACS 5-year estimates.

### Other Covariates

Based on previous studies^4,23^ and available data in the medical records, we measured age at the time of delivery (<25 years, 25-29 years, 30-34 years; ≥35 years), marital status (married vs not married), and insurance status (ever received public insurance vs never). Measures of SES in medical records were sparse, as patients provide this information voluntarily. Given a high degree of missingness for SES in the medical records, we opted not to impute the missing data because the algorithm would likely generate biased predictions. Instead, we supplemented the medical record data with the census tract median household income from the 2012-2016 ACS 5-year estimates, which has been shown to be highly associated with the residents’ actual household income.^24^ Despite the availability of health diagnosis (eg, hypertension and diabetes) and health behavior (eg, smoking) information in the medical record, we did not include such covariates in our analysis. These factors are associated with a higher risk of PTB^25,26,27^ and are likely mediators in the association between police contact and PTB; including them in the regression would have biased the association of police contact with PTB toward the null.^28^

### Statistical Analysis

First, we conducted a descriptive analysis to characterize sociodemographic characteristics, police contact levels, and PTB incidence among pregnant people in our sample. The distribution of these characteristics by racial group was compared using Pearson χ^2^ test and *t* test for categorical and continuous measures, respectively.

Second, we examined the association between police contact and PTB using logistic regression, stratified by racial group. In our unadjusted model, we regressed PTB on police contact. In the fully adjusted model, we controlled for age at the time of delivery, marital status, insurance status, and census tract median household income. For both models, we calculated odds ratios (ORs) and 95% CIs. Standard errors were calculated using the Huber-White method to account for the heteroscedasticity at the census tract level.^29^ To test the sensitivity of our regression findings, we executed similar models using a tertile-based measure of police contact.

Lastly, we tested for spatial autocorrelation between the proportion of Black residents in all Minneapolis census tracts and the cumulative police incident counts. Spatial autocorrelations were measured globally to test for clustering and locally using the Moran *I* statistics.^30^ The global measure was assessed using a Moran scatterplot and significance tested through a permutation test.^30^ For visual examination, we also created local indicator of spatial association (LISA) maps, which indicate the level of correlation between the proportion of Black residents and the police incident count in neighboring locations.^30^

All statistical tests were 2-sided, with α = .05 as the level of statistical significance. Data management and analyses of the medical record data were conducted in Stata version 13.1 (StataCorp) and R version 3.6.2 (R Project for Statistical Computing). Spatial analysis was conducted in GeoDa version 1.14.0 (Center for Spatial Data Science, University of Chicago).

## Results

Of 1059 pregnant people (745 [70.3%] White, 121 [11.4%] US-born Black, 193 [18.2%] Black born outside the US) in the sample, US-born Black individuals were younger and less likely to be married (49 [40.5%] <25 years; 27 [22.3%] married) than White individuals (37 [5.0%] <25 years; 633 [85.0%] married) and Black individuals who were born outside the US (28 [14.5%] <25 years; 146 [75.6%] married) (Table 1). Overall, 336 White pregnant people (45.1%) and 62 Black pregnant people who were born outside the US (32.1%) gave birth between the ages of 30 and 34 years. Black individuals born outside the US had sociodemographic characteristics more similar to White individuals than their US-born Black counterparts, except for their likelihood of receiving public insurance, which resembled US-born Black individuals (Black pregnant people born outside the US, 89 [46.1%]; US-born Black pregnant people, 59 [48.8%]; White pregnant people, 62 [8.3%]). Approximately half of the US-born Black individuals and Black individuals born outside the US lived in a census tract with police contact in the third tertile (US-born Black individuals, 59 [48.8%]; Black individuals born outside the US, 96 [49.7%]) compared with only 103 (13.8%) of their White counterparts. Similar police contact disparity was observed for the quartile-based police contact (fourth quartile: US-born Black individuals, 46 [38.0%]; Black individuals born outside the US, 56 [29.0%]; White individuals, 79 [10.6%]). The incidence of PTB was 6.7% for White individuals (50 pregnant people), 14.1% for US-born Black individuals (17 pregnant people), and 5.7% for Black individuals born outside the US (11 pregnant people).

**Table 1.** Cohort Descriptive Statistics, Police Contact Levels, and Incidence of Preterm Birth, Stratified by Racial Group

Characteristic	Pregnant people, No. (%)		
	White (n = 745)	Black	
		US-born (n = 121)	Born outside the US (n = 193)
Age, y			
<25	37 (5.0)	49 (40.5)	28 (14.5)
25-29	103 (13.8)	35 (28.9)	60 (31.1)
30-34	336 (45.1)	17 (14.0)	62 (32.1)
≥35	269 (36.1)	20 (16.5)	43 (22.3)
Married	633 (85.0)	27 (22.3)	146 (75.6)
Received public insurance	62 (8.3)	59 (48.8)	89 (46.1)
Household income, median (range), $	77 750 (12 895-203 036)	42 473 (12 895-104 691)	29 786 (12 895-104 691)
Residents in census tract, median, No.	3785 (1651-10 976)	3592 (1415-10 976)	3707 (1415-10 976)
Police incident per capita^a^			
Tertile 3	103 (13.8)	59 (48.8)	96 (49.7)
Quartile 4	79 (10.6)	46 (38.0)	56 (29.0)
Preterm birth	50 (6.7)	17 (14.0)	11 (5.7)

^a^ Police incident count divided by residents in census tract.

Table 2 shows the results of our regression models with the quartile-based police contact measure. In the unadjusted models comparing those who lived in census tracts with very high police contact vs their racial counterparts who lived in low-contact census tracts, we observed higher odds of PTB among White pregnant people (OR, 2.0; 95% CI, 1.9-2.0) and US-born Black pregnant people (OR, 1.5; 95% CI, 1.4-1.7) and lower odds for Black pregnant people born outside the US (OR, 0.9; 95% CI, 0.8-1.0). In the adjusted models, we observed the odds of PTB to be 90% higher for White pregnant people (OR, 1.9; 95% CI, 1.9-2.0), 100% higher for US-born Black pregnant people (OR, 2.0; 95% CI, 1.8-2.2), and 10% higher for Black pregnant people born outside the US (OR, 1.1; 95% CI, 1.0-1.2) in very high–contact census tracts vs those in low-contact census tracts.

**Table 2.** Association Between Neighborhood Quartile for Police Contact and Risk of Preterm Birth, Stratified by Racial Group

Characteristic	OR (95% CI)							
	White pregnant people (n = 745)		Black pregnant people					
	Unadjusted	Adjusted	US-born (n = 121)				Born outside the US (n = 193)	
			Unadjusted		Adjusted		Unadjusted	Adjusted
Quartile								
1-3	1 [Reference]	1 [Reference]	1 [Reference]	1 [Reference]		1 [Reference]		1 [Reference]
4	2.0 (1.9-2.0)	1.9 (1.9-2.0)	1.5 (1.4-1.7)	2.0 (1.8-2.2)		0.9 (0.8-1.0)		1.1 (1.0-1.2)
Age, y								
<25	1.1 (1.1-1.2)	NA	2.1 (1.8-2.4)	NA		2.3 (2.0-2.6)		NA
25-29	1 [Reference]	NA	1 [Reference]	NA		1 [Reference]		NA
30-34	1.2 (1.1-1.2)	NA	1.4 (1.2-1.7)	NA		1.0 (0.9-1.1)		NA
≥35	1.9 (1.9-2.0)	NA	2.7 (2.3-3.1)	NA		0.9 (0.8-1.1)		NA
Married								
No	1 [Reference]	NA	1 [Reference]	NA		1 [Reference]		NA
Yes	0.6 (0.6-0.6)	NA	1.6 (1.4-1.7)	NA		0.5 (0.5-0.6)		NA
Received public insurance								
No	1 [Reference]	NA	1 [Reference]	NA		1 [Reference]		NA
Yes	1.2 (1.2-1.3)	NA	0.9 (0.8-1.0)	NA		0.7 (0.6-0.7)		NA
Median household income	1.0 (1.0-1.0)	NA	1.0 (1.0-1.0)	NA		1.0 (1.0-1.0)		NA

Abbreviations: NA, not applicable; OR, odds ratio.

When we reclassified the police contact variable so that the census tracts in the third tertile (rather than the fourth quartile) were considered high exposure (Table 3), we observed a similar positive association between police contact and PTB for White pregnant people (OR, 1.8; 95% CI, 1.7-1.8) and US-born Black pregnant people (OR, 1.6; 95% CI, 1.4-1.8). However, the association for Black pregnant people born outside the US reversed its direction in the adjusted model (OR, 0.7; 95% CI, 0.7-0.8) (eTable 1 and eTable 2 in the Supplement).

**Table 3.** Association Between Neighborhood Tertile Police Contact and the Risk of Preterm Birth, Stratified by Racial Group

Characteristic	OR (95% CI)					
	White pregnant people (n = 745)		Black pregnant people			
	Unadjusted	Adjusted	US-born (n = 121)		Born outside the US (n = 193)	
			Unadjusted	Adjusted	Unadjusted	Adjusted
Tertile						
1 and 2	1 [Reference]	1 [Reference]	1 [Reference]	1 [Reference]	1 [Reference]	1 [Reference]
3	1.8 (1.8-1.9)	1.8 (1.7-1.8)	1.2 (1.1-1.3)	1.6 (1.4-1.8)	0.8 (0.8-0.9)	0.7 (0.7-0.8)
Age, y						
<25	1.1 (1.1-1.2)	NA	2.1 (1.8-2.4)	NA	2.3 (2.0-2.6)	NA
25-29	1 [Reference]	NA	1 [Reference]	NA	1 [Reference]	NA
30-34	1.2 (1.1-1.2)	NA	1.4 (1.2-1.7)	NA	1.0 (0.9-1.1)	NA
≥35	1.9 (1.9-2.0)	NA	2.7 (2.3-3.1)	NA	0.9 (0.8-1.1)	NA
Married						
No	1 [Reference]	NA	1 [Reference]	NA	1 [Reference]	NA
Yes	0.6 (0.6-0.6)	NA	1.6 (1.4-1.7)	NA	0.5 (0.5-0.6)	NA
Received public insurance						
No	1 [Reference]	NA	1 [Reference]	NA	1 [Reference]	NA
Yes	1.2 (1.2-1.3)	NA	0.9 (0.8-1.0)	NA	0.7 (0.6-0.7)	NA
Median household income	1.0 (1.0-1.0)	NA	1.0 (1.0-1.0)	NA	1.0 (1.0-1.0)	NA

Abbreviations: NA, not applicable; OR, odds ratio.

**Figure 2.**
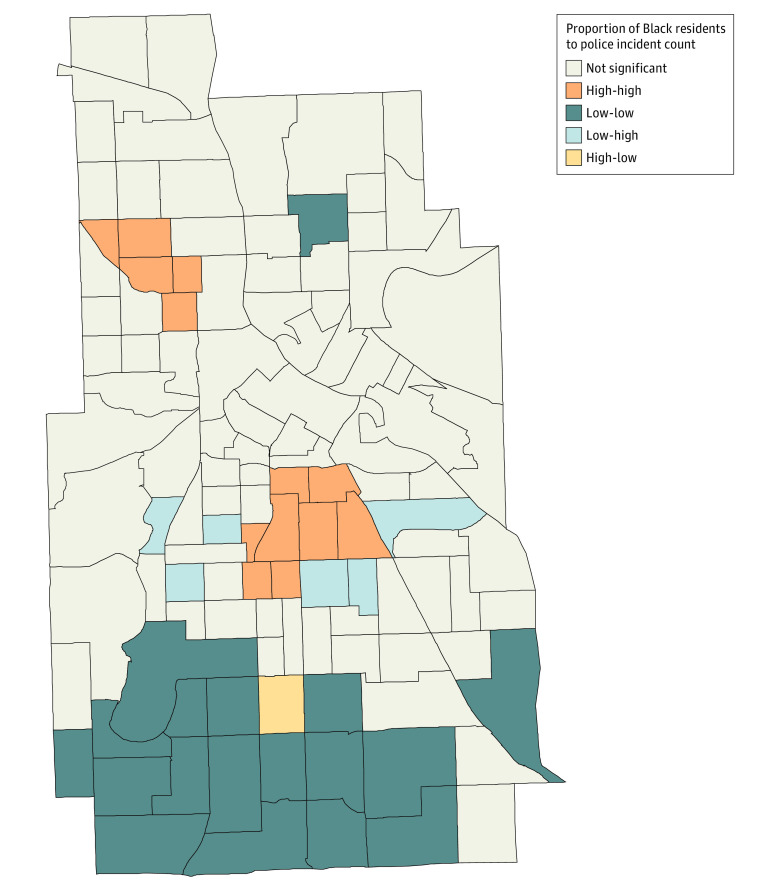
Spatial Autocorrelation for the Proportion of Black Residents and Police Incident Count by Census Tract in Minneapolis, 2012 to 2016 Figure shows clusters with a high proportion of Black residents and a high incident count (ie, high-high), those with a low proportion of Black residents and a low incident count (low-low), those with a low proportion of Black residents and high incident count (low-high), and clusters with a high proportion of Black residents and a low incident count (high-low).

Our geospatial analysis revealed a positive autocorrelation between the proportion of Black residents and the number of police incidents in the neighboring census tract with a Moran *I* of 0.237. This measure was tested with a permutation test, and clustering was significantly different than a random distribution at a pseudo-*P* value of .001. Figure 2 shows local clustering of census tracts that demonstrated a higher correlation between the proportion of Black residents and the police incident counts (the LISA statistic with *P* < .05). We observed clusters with a high proportion of Black residents and a high incident count (ie, high-high; 13 census tracts), a collection of clusters with a low proportion Black residents and a low incident count (low-low; 18 census tracts), clusters with a low proportion Black residents and high incident count (low-high; 6 census tracts), and clusters with a high proportion Black residents and a low incident count (high-low; 1 census tracts). Other clusters showed no significant clustering.

## Discussion

This study provides additional insight into the association between police contact, neighborhood context, and risk of PTB. To our knowledge, we are the first to assess the association of police contact with PTB among US-born Black pregnant people and Black pregnant people born outside the US, an important intragroup distinction. Our analyses suggest that high police contact may affect not only Black but also White pregnant people. This finding is consistent with similar studies conducted in urban populations associating racial disparities in birth outcomes with neighborhood context.^23,31,32^

Our analyses also suggest that the degree to which police contact was associated with increased risk of PTB varied for different racial groups. We found the OR of PTB was highest for US-born Black pregnant people, followed by White pregnant people and Black pregnant people who were born outside the US. The fact that we observed higher odds of PTB among White individuals further suggests that police contact acts as a stressor for everyone. Like many cities with a long history of residential segregation, the likelihood of Black people living in neighborhoods with high police contact is disproportionately high in Minneapolis. Our LISA clustering map suggested that areas in Minneapolis with high proportions of Black populations experienced disproportionately high police contact and that areas with low proportions of Black populations experienced lower police incidence. While these measures are descriptive in nature, they support an association between the racial composition of neighborhoods and the pattern of policing. Taken together, the regression and the geospatial analysis suggest that the persistent inequity in PTB between US-born Black and White pregnant people in Minneapolis could be attributable to the disproportionate policing of Black neighborhoods compared with White neighborhoods.

Black pregnant people born outside the US appeared to be an exception to the racialized exposure theory we postulated earlier. A significant proportion of the Black individuals who were born outside the US in our sample lived in neighborhoods with high police contact, but the incidence of PTB was low, even less than that of White pregnant people. We found that Black pregnant people who were born outside the US, even when living in neighborhoods with high levels of police contact, were less likely to experience PTB, even when compared with their White counterparts. These results reinforce the notion that Black identity is a social, not a biological, fact. Although both foreign- and US-born individuals occupy the same racial category (Black), the lived experience of anti-Black racism was associated with increased risk of PTB. Our findings suggest that the biological degradation associated with sustained exposure to the anti-Black racial hierarchy of the United States—which often includes cumulative and intergenerational exposures to stress inducing experiences of overpolicing—contributes significantly to the risk of PTB.

Several studies point directly to the aggressive, degrading, and racist nature of police stops and indicate that these factors make police encounters psychologically difficult for Black people to endure.^33,34,35,36^ Police contact—specifically routine stops conducted by police departments as a crime deterrent measure in certain neighborhoods—signal to Black people that in the eyes of the law, they are seen as inherently criminal and dangerous.^37^ This negative socialization coupled with the aggressive behavior that officers exhibit during these stops increases stress, anxiety, and adverse physical health for people living in these communities.^33,34,38,39^ Now more than ever, communities across the country are grappling with how to ensure public safety in light of racialized incidents of police brutality brought to their awareness in the aftermath of the murder of George Floyd, Jr, in Minneapolis, where this study took place.

### Limitations

Our study has several limitations. First, our study measured levels of police contact based on the number of police incidents in the census tract in which the pregnant people lived. However, given our ecological study design, we did not (and would not have been able to) ascertain whether the pregnant people in our study had personal contact with the police before or during pregnancy. Furthermore, it is important to note that community-level police contact is likely associated with other forms of structural racism that cannot be assessed in this study. Second, because the police incident data we used to derive the police contact measure were from before the year of birth, we made an implicit assumption that the individuals in our study had stable housing and stayed at the same address throughout the period of exposure. This was done mainly because of the cross-sectional nature of our data. The degree of housing stability likely varied for White pregnant people, US-born Black pregnant people, and Black pregnant people who were born outside the US in our sample. As a result, misclassification of the exposure variable in our analysis is possible, but it is difficult to assess given the nature of our data. Third, our sample was from 1 health system in the Minneapolis–St Paul area and may not be representative of all pregnant people in Minneapolis. Furthermore, given our small sample size, estimates from our multivariate regression may be unstable. Future research should explore the use of longitudinal data that track participants’ residence and levels of police contact dynamically to address these limitations. We also recommend the use of data from other representative sources (eg, restricted vital statistic data with geocoding variable) with a larger sample size to assess whether the association reported in our study is true in other locales, especially in urban areas with high proportions of Black immigrants (eg, New York City; Washington, DC; Seattle). Additionally, not all types of police contact may carry the same stress-inducing effects and may be associated with the odds of PTB to at a different degree. Future studies should examine the potential heterogeneous associations of police contact with health outcomes by offense type.

## Conclusions

In conclusion, we found that police contact was associated with increased risk of PTB among White pregnant people, US-born Black pregnant people, and Black pregnant people who were born outside the US. However, because neighborhoods with a greater proportion of Black residents were more likely to be policed, the higher incidence of PTB among Black pregnant people than White pregnant people may be attributed to racialized exposure rather than a differential effect of police contact between racial groups. These findings suggest that racialized police patterns borne from a history of structural racism in the United States may contribute to racial disparity in PTB.

## Notes

Supplement. **eFigure. **Sample Inclusion **eTable 1. **Multivariate Logistic Regression Testing the Significance of Racial Group as an Effect Modifier for Police Contact (Quartile 4 vs Quartile 1-3) **eTable 2.** Multivariate Logistic Regression Testing the Significance of Racial Group as an Effect Modifier for Police Contact (Tertile 3 vs Tertile 1 and 2)
