# Supplementary material for: Association of Residence in High–Police Contact Neighborhoods With Preterm Birth Among Black and White Individuals in Minneapolis
Source: JAMA Netw Open. 2021 Dec 8;4(12):e2130290. doi: 10.1001/jamanetworkopen.2021.30290 (PMC8655601; doi:10.1001/jamanetworkopen.2021.30290)
Supplement: Supplement. — eFigure. Sample Inclusion eTable 1. Multivariate Logistic Regression Testing the Significance of Racial Group as an Effect Modifier for Police Contact (Quartile 4 vs Quartile 1-3) eTable 2. Multivariate Logistic Regression Testing the Significance of Racial Group as an Effect Modifier for Police Contact (Tertile 3 vs Tertile 1 and 2) [file jamanetwopen-e2130290-s001.pdf]

Supplemental Online Content

Hardeman RR, Chantarat T, Smith ML, Karbeah JM, Van Riper DC, Mendez DD. Association of residence in high–police contact neighborhoods with preterm birth among Black and White individuals in Minneapolis. *JAMA Netw Open.* 2021;4(12):e2130290. doi:10.1001/jamanetworkopen.2021.30290

eFigure. Sample Inclusion

eTable 1. Multivariate Logistic Regression Testing the Significance of Racial Group as an Effect Modifier for Police Contact (Quartile 4 vs Quartile 1-3)

eTable 2. Multivariate Logistic Regression Testing the Significance of Racial Group as an Effect Modifier for Police Contact (Tertile 3 vs Tertile 1 and 2)

This supplemental material has been provided by the authors to give readers additional information about their work.

© 2021 Hardeman RR et al. *JAMA Network Open.*

eFigure. Sample Inclusion

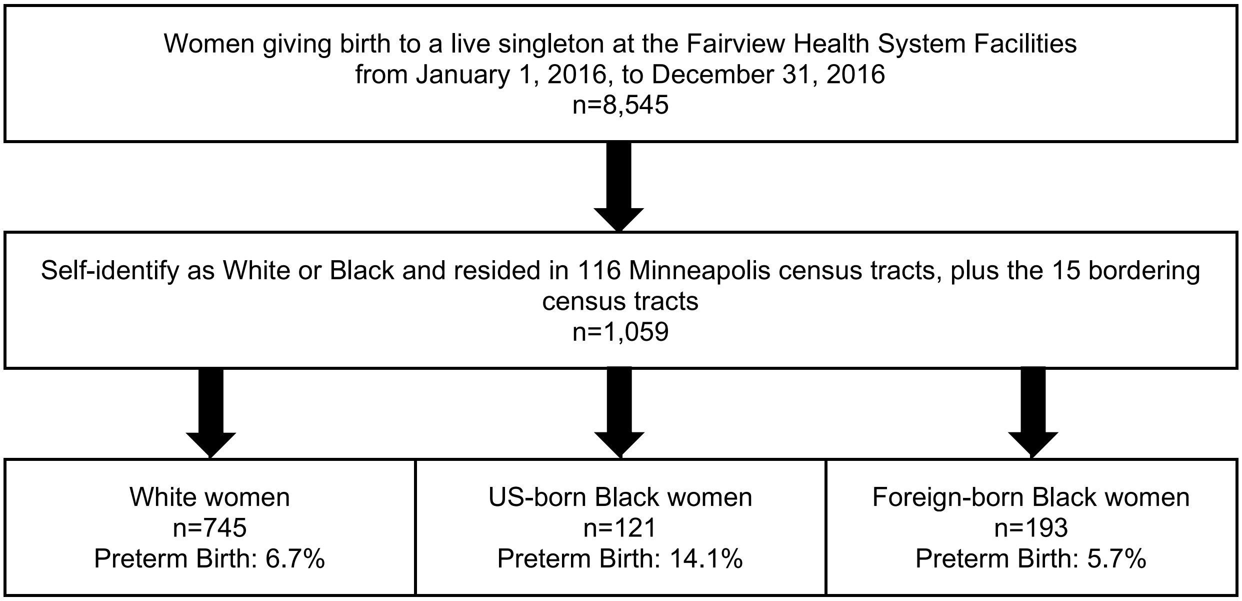

© 2021 Hardeman RR et al. *JAMA Network Open.*

eTable 1. Multivariate Logistic Regression Testing the Significance of Racial Group as an Effect Modifier for Police Contact (Quartile 4 vs. Quartile 1-3)

	Beta	SE	*P* Value
Intercept	-2.832	0.016	<0.001
Police contact in quartile 4	0.688	0.013	<0.001
US-born Black	0.648	0.015	<0.001
Foreign-born Black	0.015	0.014	0.27
Younger than 25 years old	0.476	0.014	<0.001
30-34 years	0.267	0.013	<0.001
35 years and older	0.729	0.012	<0.001
Married	-0.319	0.011	<0.001
Publicly insured	-0.099	0.011	<0.001
Median household income	0.000	0.000	<0.001
Police contact in quartile 4*US-born Black	-0.359	0.020	<0.001
Police contact in quartile 4*Foreign-born Black	-0.824	0.025	<0.001

[a] Huber-White standard errors

© 2021 Hardeman RR et al. *JAMA Network Open.*

eTable 2. Multivariate Logistic Regression Testing the Significance of Racial Group as an Effect Modifier for Police Contact (Tertile 3 vs. Tertile 1&2)

	Beta	SE[a]	*P* Value
Intercept	-2.852	0.017	<0.001
Police contact in tertile 3	0.610	0.012	<0.001
US-born Black	0.736	0.016	<0.001
Foreign-born Black	0.105	0.016	<0.001
Younger than 25 years old	0.505	0.014	<0.001
30-34 years	0.264	0.013	<0.001
35 years and older	0.724	0.012	<0.001
Married	-0.308	0.011	<0.001
Publicly insured	-0.127	0.011	<0.001
Median household income	0.000	0.000	<0.001
Police contact in tertile 3*US-born Black	-0.473	0.020	<0.001
Police contact in tertile 3*Foreign-born Black	-0.833	0.022	<0.001

[a] Huber-White standard errors

© 2021 Hardeman RR et al. *JAMA Network Open.*
